# Dynamic Transcriptome Analysis Reveals Complex Regulatory Pathway Underlying Induction and Dose Effect by Different Exogenous Auxin IAA and 2,4-D During *in vitro* Embryogenic Redifferentiation in Cotton

**DOI:** 10.3389/fpls.2022.931105

**Published:** 2022-06-29

**Authors:** Yupeng Fan, Zhengmin Tang, Junmei Wei, Xiaoman Yu, Huihui Guo, Tongtong Li, Haixia Guo, Li Zhang, Yijie Fan, Changyu Zhang, Fanchang Zeng

**Affiliations:** ^1^State Key Laboratory of Crop Biology, College of Agronomy, Shandong Agricultural University, Tai'an, China; ^2^College of Life Sciences, Huaibei Normal University, Huaibei City, China

**Keywords:** cotton, somatic embryogenesis, embryogenic redifferentiation, indole acetic acid (IAA), 2, 4-dichlorophenoxyacetic acid (2, 4-D), embryogenic induction effect, dose effect, transcriptional regulation

## Abstract

Plant somatic cells can reprogram into differentiated embryos through somatic embryogenesis (SE) on the condition of plant growth regulators (PGRs). RNA sequencing analysis was performed to investigate transcriptional profiling on cotton redifferentiated callus that was induced by different auxin types (IAA and 2,4-D), different concentrations (0, 0.025, and 0.05 mg L^−1^), and different incubation times (0, 5, and 20 days). Under the 2,4-D induction effect, signal transduction pathways of plant hormones were significantly enriched in the embryogenic response stage (5 days). These results indicated that auxin signal transduction genes were necessary for the initial response of embryogenic differentiation. In the pre-embryonic initial period (20 days), the photosynthetic pathway was significantly enriched. Most differentially expressed genes (DEGs) were downregulated under the induction of 2,4-D. Upon the dose effect of IAA and 2,4-D, respectively, pathways were significantly enriched in phenylpropanoid biosynthesis, fatty acid metabolism, and carbon metabolic pathways. Therefore, primary and secondary metabolism pathways were critical in cotton SE. These results showed that complex synergistic mechanisms involving multiple cellular pathways were the causes of the induction and dose effect of auxin-induced SE. This study reveals a systematic molecular response to auxin signals and reveals the way that regulates embryogenic redifferentiation during cotton SE.

## Introduction

Plant cells reserve totipotency competence, allowing various explants to regenerate into whole plants in culture conditions *in vitro*. Somatic embryogenesis (SE) is the potential ability of the somatic cell to regenerate into seedlings under artificial conditions, and it is a remarkable example of plant cell totipotency. SE is useful in many fields, including plant regeneration, genetic engineering, development, and utilization of plant resources. In different species, such as carrot, Arabidopsis, soybean, and potato, the molecular mechanisms of SE have been described (Toonen et al., 1994; Thibaud-Nissen et al., 2003; Kurczynska et al., 2007; Sharma et al., 2008). The plant segments transform into differentiation calli, and then the calli regenerate into somatic embryos. Scientists have obtained regeneration plants through SE. Stress and plant hormones are the two most important factors involved in the initiation of SE (Karami and Saidi, 2010). However, the potential molecular mechanisms of transformation from somatic cells to embryogenesis ability cells are of great significance for revealing theoretical problems and solving practical applications (Zimmerman, 1993; Chugh and Khurana, 2002; Zeng et al., 2007a; Sakhanokho and Rajasekaran, 2016). Therefore, it is crucial to have an insight into the molecular mechanisms of embryogenesis and development.

Cotton is an important commercial crop globally, which supplies fiber for people worldwide, such as all kinds of textiles (Wilkins et al., 2000). In modern cotton molecular engineering, genotype dependency is a limited step in transgenic development (Zhang et al., 1991; Mishra et al., 2003; Ganesan and Jayabalan, 2004). Of the existing cultivated cotton varieties, only a few have a high regeneration ability, such as YZ-1 (Jin et al., 2006); the others are scarce, even recalcitrant to regenerate. The genotype dependence hinders the biological technology utilization in cotton. Cotton is one of the problematic plants to regenerate (Scowcroft, 1984).

Many factors affect cotton SE, such as culture media composition, plant growth regulators (PGRs), explants, and environmental conditions (Sakhanokho and Rajasekaran, 2016). Hormones and stress are two main reasons concerning the initiation of cell dedifferentiation and plant embryogenesis by regulating various gene expressions (Feher et al., 2003; Ikeda-Iwai et al., 2003; Rose and Nolan, 2006). The SE process is traditionally divided into two stages, namely, induction and expression. In general, induced tissues or cells reach the expression stage by *in vitro* culture, then, the cells show their embryogenesis ability and differentiate into somatic embryos. SE induction stimulated by exogenous PGRs is a complicated multifactorial system (Jimenez, 2005). SE depends on complex interactions between different PGRs, including auxin alone or auxin combined with cytokinin during the initial pro-embryogenic phase. In most species studies, PGRs are essential to induce SE, and auxin and cytokinin are critical factors in the SE process, probably because they are involved in cell division and cell cycle regulation (Kumar and Van Staden, 2017).

Plant hormones are naturally organic signaling molecules that play critical roles in coordinating all aspects of plant growth, development, and stress adaptations at low concentrations. Signal transduction of exogenous plant hormones is carried out through several distinctive approaches in tissue culture. The study on tissue cultures of major grain and economic crops is a systematic study that explores the phytohormones' inductive and regulating functions in the process of SE (Li and Li, 2019), including auxin and cytokinin. Auxin is considered to be the most critical hormone regulating SE *in vitro* (Cooke, 1993). Auxin regulates the development of plant embryos. Su et al. (2009) found that auxin can induce expression of the *Wuschel (WUS)* gene which is very important for the regeneration of embryonic stem cells during the SE process. Cheng et al. (2013) found that auxin response factor3 (*ARF3*) could bind to the promoter of cytokinin synthase gene *IPT5* and inhibit its expression, thus inhibiting the biosynthesis of cytokinin. Su's research (Su et al., 2009) showed that the auxin gradients and polarity distribution were extremely important for *WUS* regulation during SE induction. The polar transport activity of auxin buffered the normal distribution of auxin, and *PIN* was the most critical gene (Weijers et al., 2005; Su et al., 2009). It is reported that explants in a medium with the supplementation of 2,4-dichlorophenoxyacetic acid (2,4-D) increase the level of endogenous auxin in reactive explants. The endogenous auxin makes one of the syntheses of the critical signal to determine the fate of cultured cells' embryogenesis (Thomas et al., 2002). The balance between cytokinin and auxin is essential for all development processes. During the course of auxin signal transduction, *Aux/IAAs* combined with *ARFs* inhibited the activity of *ARF* under low auxin concentration (Salehin et al., 2015). However, in the process of embryo regeneration, the interaction between genes in the auxin and cytokinin signal transduction pathway has not been fully understood.

The somatic cell reenters the cell cycle and develops into a complete plant, during this process many genes are involved. Some candidate genes, specifically SE-related genes, have been identified, including *auxin response factor* (*ARF*) (Su et al., 2016), *leafy cotyledon* (*LEC*) (Rupps et al., 2016), *baby boom* (*BBM*) (Rupps et al., 2016), *WUS* (Elhiti et al., 2010), and *somatic embryogenesis receptor kinase* (*SERK*) (Hu et al., 2005), which play a decisive role in SE (Kumar and Van Staden, 2017; Guo et al., 2020). Extensive research has investigated physiological and biochemical changes in various plants in the process of SE, emphasizing understanding the gene regulation mechanisms related to SE. Scientists have identified DEGs in somatic embryos (SEs), emphasized the possible pathways involved in SE, and found protein or molecular markers in SE (Mantiri et al., 2008). Some classic genes have been proven to play a decisive role in SE in experiments. In the hypocotyl regeneration of carrots, Schmidt et al. (1997) screened an important gene named *SERK1*, which transformed somatic cells into embryonic cells. Since then, a series of genes have been discovered by scientists, such as *LEC1, LEC2, FUS3*, and *AGL15* (Lotan et al., 1998; Stone et al., 2001, 2008; Harding et al., 2003), which are induced in embryogenic callus during SE and further regulate downstream physiological processes to control embryo development. The pathways to auxin and cytokinin signals play a critical role in the dedifferentiation and redifferentiation of somatic cells in cotton (Zeng et al., 2007b; Yang et al., 2012; Xu et al., 2013; Min et al., 2015). Auxin and cytokinin are primary growth regulators that are relevant to cell differentiation and division for plants. The cotton seedlings can be regenerated through the SE process in the medium with various combinations of PGRs, such as indole-3-butyric acid (IBA), naphthalene acetic acid (NAA), 2,4-dichlorophenoxyacetic acid (2,4-D), and combined with kinetin (KT) (Sun et al., 2006). Global transcriptome analysis shows that auxin and cytokinin signaling pathways were vital in cotton somatic cells' dedifferentiation and redifferentiation. In addition, the genes and pathways of the stress response are also found to be related to SE (Yang et al., 2012). The *ERF* plays a key role in hormone signaling and the connection of different hormone pathways (Vogler and Kuhlemeier, 2003). Jasmonic acid can rapidly induce the expression of *ERF109* and activate *CYCD6;1* transcription and an injury signal induces local accumulation of auxin and activates *CYCD6;1*, which plays a key role in plant tissue regeneration (Zhang et al., 2019; Zhou et al., 2019). Despite these studies, the molecular mechanisms underlying cotton SE are still unknown.

Cotton genome information has been continuously improved (Patel and Jain, 2012; Wang et al., 2012) with the progress of the next-generation sequencing technology. Scientists have discovered more than 5,000 differential expression genes with Digital Gene Expression (DGE) technology in cotton SE. Scientists have discovered that auxin is involved in this process (Yang et al., 2012). At the same time, several small RNAs and their targets were discovered in somatic embryos by sRNA-seq and degradome sequencing. The appearance of differential expression (Yang et al., 2013) shows that SE in cotton is regulated by complicated gene expression. Our research work aims to unearth many SE-related genes with critical research value, which will help to elucidate the molecular mechanism of SE and point out the direction for studying the mechanism of SE. Our chief tactics were to use the transcriptome sequencing technology of the YZ-1 genotype to make out the DEGs expressed in the SE induction, and YZ-1 material has been proved to be highly embryogenic under the conditions of SE induction culture. The acquisition of embryonic regenerative capacity is primarily associated with cell dedifferentiation, from the current developmental pathway to the response to phytohormone stimulation signals and cell division by targeting embryogenic cells. In the early stage of embryogenesis, a series of changes occur to somatic cells, including cell dedifferentiation, induction of embryogenic callus, and acquisition of embryonic regeneration ability. During the processes of cell embryogenesis and plant regeneration, there is heterogeneity in different environments. The environmental manifestation of genotype heterogeneity is not merely a genotype variation but a genotype and environmental interaction-induced variation. Representing factor response, the phenotype is a result of an interaction between genotype and environmental conditions. Furthermore, the process of SE and plant regeneration is regulated by intracellular genetic factors and extracellular environment-inducing factors, especially the regulation of exogenous growth regulator inducers.

Plant SE has been extensively studied from the perspectives of cell levels and physiological, biochemical, and molecular levels. Understanding the molecular mechanisms of different plant genetic resources helps improve the embryogenesis ability of other plant species and discover new methods of plant breeding and crop improvement. This study will be beneficial in understanding the molecular mechanism of SE, especially the continuous development of sequencing technology that will reveal the secret of somatic cells developing into pluripotent cells. Our principal tactics were to identify genes differentially expressed in the SE induction using transcriptome sequencing technology. The plant material was YZ-1, selected from highly embryogenic upland cotton species. Different auxins (IAA and 2,4-D) combined with KT were added to the medium.

## Materials and Methods

### Plant Materials and Cultural Conditions

The upland cotton YZ-1 (*Gossypium hirsutum* L.) with high SE ability was used for the study. First, we soaked YZ-1 seeds with a 0.1% (w/v) HgCl_2_ solution for 10 min, then washed the seeds with sterile water four times, and germinated on 1/2 MS medium with 1.5% (w/v) sucrose and 0.25% (w/v) phytagel. The pH of the medium was 6.0. Calli were induced from 5 to 7 mm hypocotyl sections of 5–7-day-old seedlings, as described by Wu et al. (2004). The dedifferentiation medium was MS medium with the addition of 0.1 mg L^−1^ 2,4-D (Sigma, Co., St. Louis, MO, USA) and 0.1 mg L^−1^ KT (Sigma, Co., St. Louis, MO, USA), and 0.1 mg L^−1^ 2,4-D was marked as C3 in the description of 24D-C3-0D. After 6 weeks of cultivation, as described by Zeng et al. (2007a), the callus was isolated and transferred into a redifferentiation medium supplemented with 0.1 mg L^−1^ KT and various exogenous auxins (IAA and 2,4-D) to induce somatic cells. A series of concentration gradient treatments of exogenous auxin was set as 0 mg L^−1^ (C0), 0.025 mg L^−1^ (C1), and 0.05 mg L^−1^ (C2). Primary callus was collected at 0, 5, and 20 days after callus redifferentiation induction. The embryogenic response stage (5 days) and pre-embryonic initial period (20 days) were defined based on cytochemical analysis of cell morphology and embryogenic competence in cotton cell culture (Guo et al., 2019c). Three biological replicates were set in all experimental treatments.

### Library Construction and RNA-Sequencing

RNA extraction was conducted according to the EASYspin Plus plant RNA rapid extraction kit instructions (Aidlab Biotechnologies Co. Ltd., Beijing, China). The sequencing libraries were prepared by the NEBNext^®^ Ultra™ RNA Library Prep Kit (Illumina, San Diego, CA, USA). The cDNA libraries were sequenced on the Illumina HiSeq platform (GENEWIZ Biotechnology Co., Ltd, Suzhou, China). The sequencing strategy was PE-150. Bioinformatic analysis was performed based on RNA-Seq from a series of sequenced samples. The RNA-Seq data mining and analysis have been completed in our laboratory.

### Reads Mapping, Enrichment of GO, and KEGG Analysis

The upland cotton reference genome (https://www.cottongen.org/species/Gossypium_hirsutum/nbi-AD1_genome_v1.1) was used as the reference genome. The Hisat2 (v2.0.1) software compared the filtered sequencing clean data with a reference genome. We used RPKM to calculate the gene expression levels of different comparison groups. Read counts were standardized by DESeq2 (v1.6.3, http://www.bioconductor.org/) (Anders and Huber, 2010). DEGs were defined as that ≥ 2-fold change and FDR ≤ 0.05. Three replicates of standardized sequencing data with repeatability were applied for analysis (He et al., 2013).

GO terms of biological process, molecular function, and cellular component were described by GO-Term Finder (v0.86, http://search.cpan.org/dist/GO-TermFinder/). GO terms with a *p*-value < 0.05 were considered significant. The KEGG database was used to determine which pathways were enriched by the in-house script (http://en.wikipedia.org/wiki/KEGG).

### RNA-Seq Data Validation by qRT-PCR

Three replicates of qRT-PCR were performed for each sample to verify the DEGs detected by the transcriptome sequencing. We selected six genes for validation and analysis. We used Primer Premier 5.0 software (http://www.premierbiosoft.com/primerdesign/) to design primers (Table A1). Primers were synthesized in the Shanghai Shenggong Company. We synthesized cDNA according to the manufacturer's instructions using EasyScript One-Step gDNA Removal and cDNA Synthesis SuperMix (TransGen Biotech, Beijing, China). QRT-PCR was performed in 15 μl reactions on the Real-Time Thermal Cycler (Analytik Jena AG, qTOWER^3^G, Germany). The template was 1.5 μl cDNA of the first strand. The rest were 7.5 μl of 2 × UltraSYBR Mixture (with ROX) (CWBIO, Beijing, China), 1.2 μl 10 μM forward primer, 1.2 μl 10 μM reverse primer, and 3.6 μl of ddH_2_O. The reference gene was *GhUB7*. The qRT-PCR program was set as follows: preincubation at 95°C, 10 min first; the second step was amplification at 95°C for 15 s, 60°C for 30 s, and 72°C for 30 s, 38 cycles in total. A melting curve analysis was performed in order to evaluate the primer's specificity. The melting curve analysis program was set as follows: 95°C for 15 s, 60°C for 60 s, 95°C for 15 s, and 60°C for 15 s.

## Results

### Callus Differentiation Rate Induced by Various Hormone Combinations Differed in Cotton

On MS medium with 0.1 mg L^−1^ 2,4-D and 0.1 mg L^−1^ KT (0.1 mg L^−1^ was named C3 in the 24D-C3-0D),5–7 mm hypocotyl segments were cultured. After 6 weeks of cultivation, we transferred calli into the medium with different auxin concentrations to induce embryogenic calli. After 30 days of cultivation, we calculated the embryogenic callus induction rate. Statistics showed that the differentiation rate of callus was the lowest on the medium with 0.1 mg L^−1^ KT alone. After adding different auxins (IAA, 2,4-D) based on 0.1 mg L^−1^ KT, the differentiation rate of callus apparently increased (Table 1). The combined utilization of auxin and KT in cotton callus differentiation is essential.

**Table 1:** Statistics of callus differentiation rate of cotton YZ-1.

**Types of auxin**	**Auxin concentration (mg L^**−1**^)**	**KT Concentration (mg L^**−1**^)**	**Callus differentiation rate (%)**
IAA	0.05	0.1	70.83
IAA	0.025	0.1	42.86
2,4-D	0.05	0.1	33.82
2,4-D	0.025	0.1	19.44
–	0	0.1	7.81

### Differentially Expressed Genes (DEGs) Screened Were Different Between Samples

**Figure 1:**
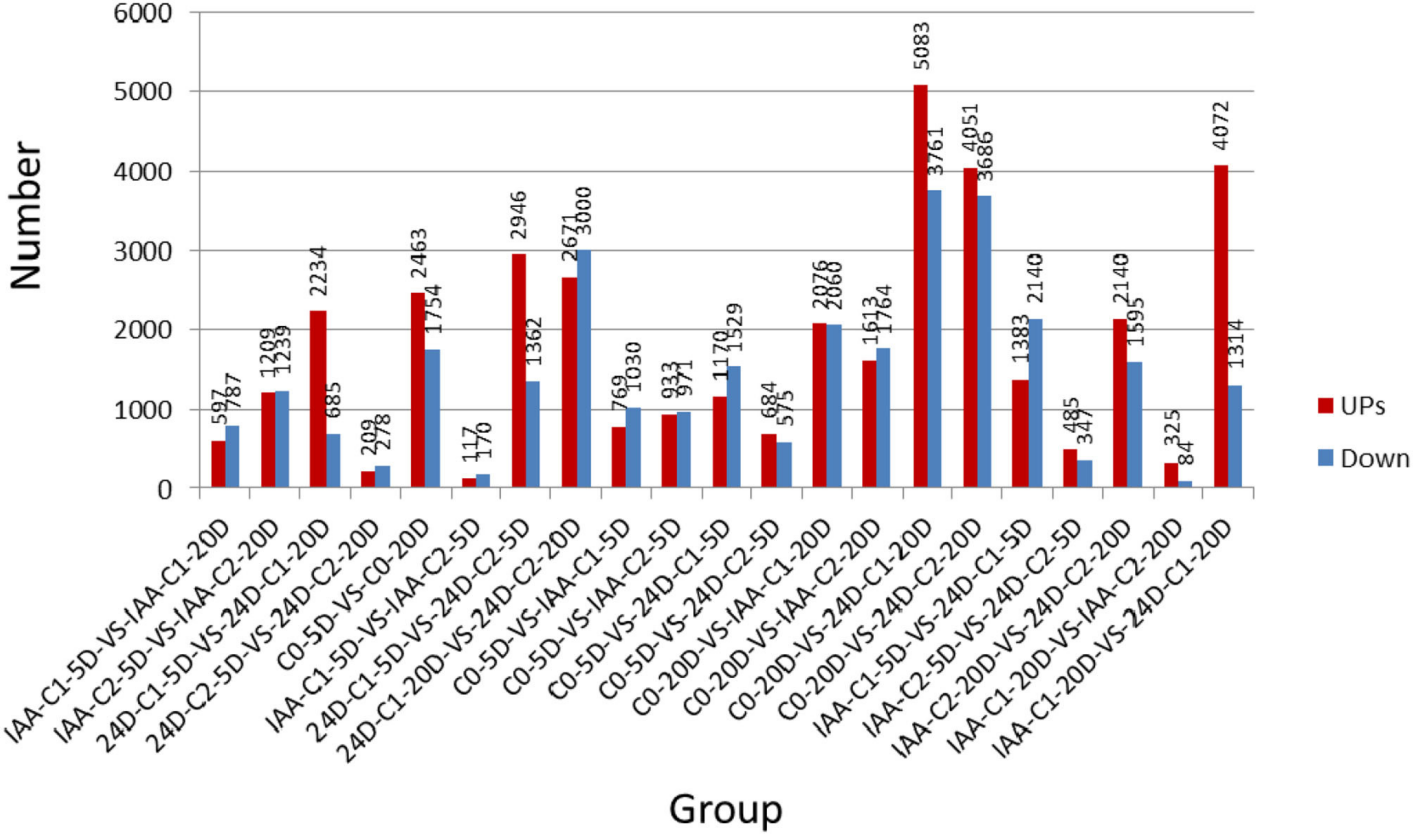
The number of upregulated and down-regulated DEGs was compared between samples. IAA, indole acetic acid; 24D, 2,4-dichlorophenoxyacetic acid; C0, 0 mg L^−1^; C1, 0.025 mg L^−1^; C2, 0.05 mg L^−1^; 5D, 5 days' treatment; 20D, 20 days' treatment.

The expression level was used for the subsequent analysis. Reads per kilo base per million reads (RPKM) were applied to calculate the gene expression level. The gene length and sequence depth were also standardized, so gene expression levels with different sequence depths and different lengths could be compared with each other. The number of upregulated and downregulated DEGs was compared between samples (Figure 1).

### Sequencing Annotation Analysis Statistics of Different Transcriptome Comparisons in Various Databases

During the process of cotton somatic embryogenesis (SE), the transcriptome was sequenced and analyzed using high-throughput sequencing technology. We have found that 284–8,627 genes were aligned to the GenBank non-redundant protein sequence (NR) database, 216–5,756 genes were aligned to the Gene Ontology (GO) database, and 105–2,387 genes were aligned to the Kyoto Encyclopedia of Genes and Genomes (KEGG) database (Table 2).

**Table 2:** Summary of annotated genes in each database of the pairwise comparisons.

**Pairwise comparisons**	**NR**	**GO**	**KEGG**
IAA-C1-5D VS IAA-C1-20D	1,347	943	327
IAA-C2-5D VS IAA-C2-20D	2,369	1,640	587
24D-C1-5D VS 24 D-C1-20D	2,865	2,006	748
24D-C2-5D VS 24D-C2-20D	465	323	99
C0-5D VS C0-20D	4,118	2,785	1,191
IAA-C1-5D VS IAA-C2-5D	284	216	105
IAA-C1-20D VS IAA-C2-20D	384	242	96
24D-C1-5D VS 24D-C2-5D	4,154	2,858	994
24D-C1-20D VS 24D-C2-20D	5,530	3,789	1,296
C0-5D VS IAA-C1-5D	1,742	1,197	362
C0-5D VS IAA-C2-5D	1,823	1,280	474
C0-5D VS 24D-C1-5D	2,604	1,813	682
C0-5D VS 24D-C2-5D	1,204	795	294
C0-20D VS IAA-C1-20D	4,021	2,787	1,140
C0-20D VS IAA-C2-20D	3,275	2,250	1,005
C0-20D VS 24D-C1-20D	8,627	5,756	2,387
C0-20D VS 24D-C2-20D	7,543	5,110	2,065
IAA-C1-5D VS 24D-C1-5D	3,424	2,436	826
IAA-C1-20D VS 24D-C1-20D	5,252	3,563	1,318
IAA-C2-5D VS 24D-C2-5D	785	513	170
IAA-C2-20D VS 24D-C2-20D	3,606	2,461	832

*IAA, indole acetic acid; 24D, 2,4-dichlorophenoxyacetic acid; C0, 0 mg L^−1^; C1, 0.025 mg L^−1^; C2, 0.05 mg L^−1^; 5D, 5 days' treatment; 20D, 20 days' treatment*.

### Screening DEGs From Venn Diagram Among Different Comparison Groups

**Figure 2:**
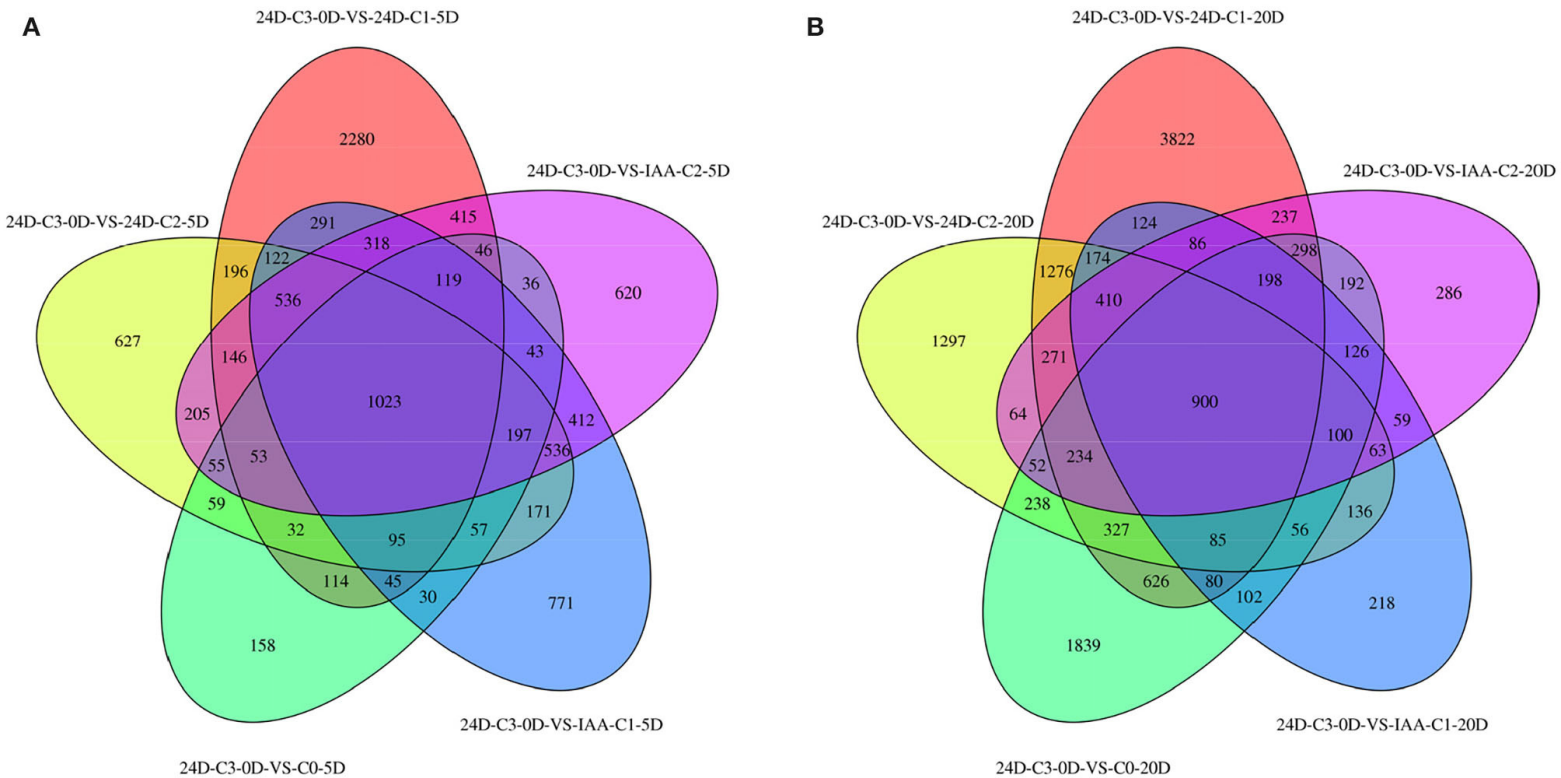
Venn diagram of DEGs between different comparison groups. **(A)** C3 was compared with 0.025 mg L^−1^ of IAA at 5 days. **(B)** C3 was compared with different concentrations of 2,4-D. IAA, indole acetic acid; 24D, 2,4-dichlorophenoxyacetic acid; C0, 0 mg L^−1^; C1, 0.025 mg L^−1^; C2, 0.05 mg L^−1^; C3, 0.1 mg L^−1^; 5D, 5 days' treatment; 20D, 20 days' treatment.

We sampled cotton callus for transcriptome analysis and found that when C0 was compared with IAA and 2,4-D treatments, 1,023 and 900 genes were expressed in common in the two comparison groups (Figure 2). It indicated that the number of genes expressed in common in 5 days was more than that expressed in 20 days.

Comparing the number of specifically expressed genes from the Venn diagram (Figure 2) in the embryogenic activating stage (5 days), the lower IAA concentration (0.025 mg L^−1^) induced more specifically expressed genes, but the callus differentiation rate was lower (42.86 vs. 70.83%). The lower 2,4-D concentration (0.025 mg L^−1^) induced more specifically expressed genes (nearly three times), but the callus differentiation rate was lower (19.44 vs. 33.82%) too. These results showed that excessive genes activated in lower IAA and 2,4-D concentrations, but it was not conducive to embryogenesis (5 days). A proper and consistent gene expression was helpful for differentiation.

At 0.025 mg L^−1^ of IAA and 2,4-D, the specifically expressed genes induced by 2,4-D are more than that induced by IAA. Compared to the phenotype, the differentiation effect of IAA was better than that of 2,4-D, which indicated that the more specifically expressed genes adversely affected callus differentiation in the initial stage of differentiation (5 days).

However, at the 0.05 mg L^−1^ concentration, there was less of a difference between the number of specific genes induced by IAA and 2,4-D. It was speculated that there was a different embryogenic induction mechanism between IAA and 2,4-D at the embryogenic responsive stage.

In the pre-embryonic initial stage (20 days), the number of specifically expressed genes induced by 2,4-D increased steadily. But the number of specifically expressed genes induced by IAA apparently decreased. There was almost no difference between the amounts of specific genes induced by different concentrations of IAA. The different callus differentiation rates indicated that there were not many specific expressed genes involved in the callus differentiation induction upon the pre-embryonic initial stage. The more specifically expressed genes induced by 2,4-D, there was no conducive to callus differentiation (20 days). It was speculated that with the extension of culture time, callus can adjust to different concentrations of IAA. Moreover, the number of specific genes induced in the embryogenic activating stage (5 days) was more crucial.

### GO Functional Annotation of Identified DEGs

By applying different concentration gradients of auxin, GO functional annotation of DEGs was conducted in cotton SE. In the GO functional classification system, all the comparison groups were related to biological processes, cell components, and molecular function. In “molecular function” GO terms, significant enrichments were found in protein binding, nucleic acid binding, transcription factor activity, transporter activity, and catalytic activity. In “cellular component” GO terms, significant enrichments were located in the cell part, membrane, organelle, and membrane part. In “biological process” GO terms, the secondary GO annotation terms were significantly enriched in cellular process, localization, biological regulation, and metabolic process. The gene expression patterns were similar, induced by different auxin concentrations.

**Figure 3:**
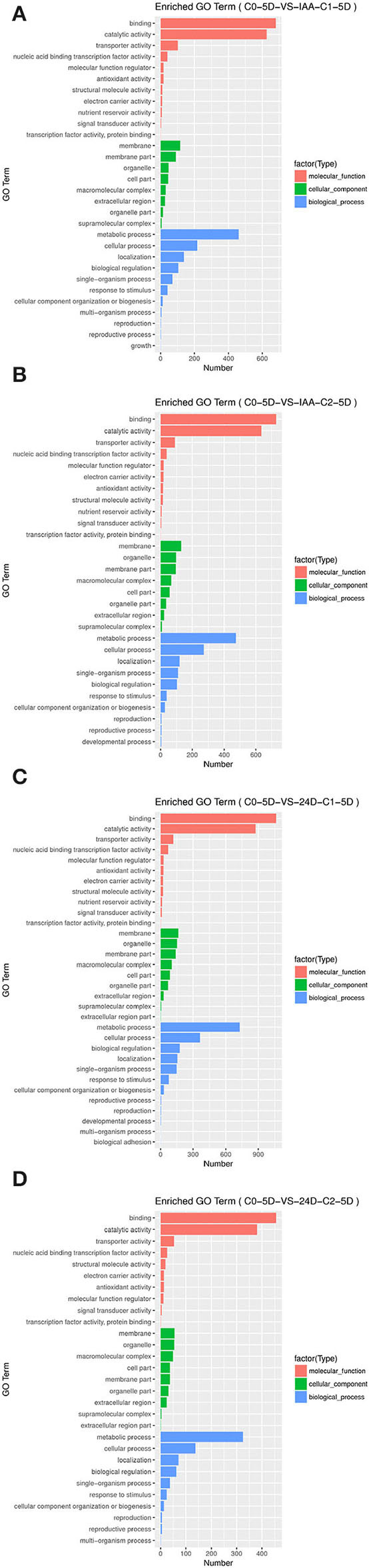
Histogram of GO enrichment of DEGs from samples treated with different auxin concentrations. **(A)** Enriched GO terms in C0-5D vs. IAA-C1-5D; **(B)** Enriched GO terms in C0-5D vs. IAA-C2-5D; **(C)** Enriched GO terms in C0-5D vs. 24D-C1-5D; **(D)** Enriched GO terms in C0-5D vs. 24D-C2-5D. IAA, indole acetic acid; 24D, 2,4-dichlorophenoxyacetic acid; C0, 0 mg L^−1^; C1, 0.025 mg L^−1^; C2, 0.05 mg L^−1^; 5D, 5 days' treatment.

However, the number of differential inductive genes varied enormously (Table 2). In a comparison of different concentrations of auxin in groups C0-5D vs. IAA-C1-5D and C0-5D vs. IAA-C2-5D (Figures 3A,B), C0-5D vs. 24D-C1-5D, and C0-5D vs. 24D-C2-5D (Figures 3C,D), when IAA was added, there was no apparent difference in the number of DEGs with the rise of concentration; when 2,4-D was supplemented, the amount of DEGs decreased in higher concentration.

For the number of DEGs in the comparison group C0-5D vs. IAA-C1-5D (Figure 3A), C0-5D vs. 24D-C1-5D (Figure 3C), with the same concentration of different auxins, the number of DEGs induced in 24D-C1-5D was more than that induced in IAA-C1-5D. For the number of DEGs in the comparison group C0-5D vs. IAA-C2-5D (Figure 3B), and C0-5D vs. 24D-C2-5D (Figure 3D), with the same concentration of different auxins, the number of DEGs induced in IAA-C2-5D was more than that induced in 24D-C2-5D. It indicates that the induction effect was different in different concentrations of IAA and 2,4-D, respectively.

### KEGG Enrichment Specific Pathway Analysis

Auxin significantly affected the embryogenic differentiation of cotton callus (Table 1). We analyzed the DEGs related to the embryogenic differentiation rate of cotton callus induced by auxin. The induction effect was different from the comparisons between with and without auxin. We chose the auxin concentration to be consistent with the higher differentiation rate. The cotton callus embryogenic differentiation rate induced by auxin concentrations was also different. The dose effect indicated that the different concentrations were compared, including 0 mg L^−1^ (C0) vs. 0.025 mg L^−1^ (C1), and 0.025 mg L^−1^ (C1) vs. 0.05 mg L^−1^ (C2).

#### KEGG Enrichment Pathway Induced by IAA

##### Enriched Specific Pathway Under IAA Induction Effect

**Figure 4:**
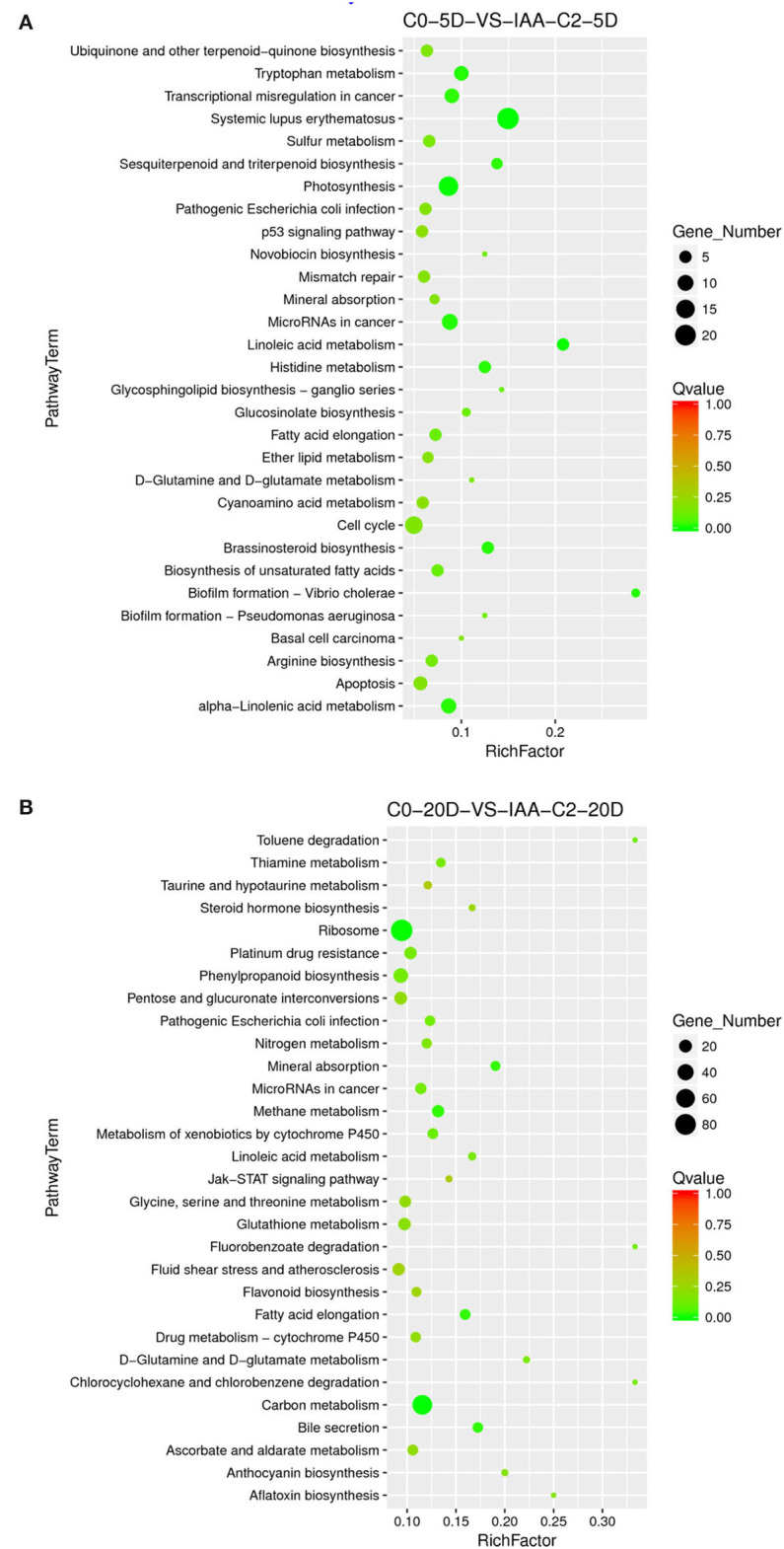
The KEGG enrichment of DEGs in comparison groups under the IAA induction effect. **(A)** Pathways enriched in C0-5D vs. IAA-C2-5D; **(B)** Pathways enriched in C0-20D vs. IAA-C2-20D. IAA, indole acetic acid; C0, 0 mg L^−1^; C2, 0.05 mg L^−1^; 5D, 5 days' treatment; 20D, 20 days' treatment.

The results of the differentiation rate showed that auxin had an extraordinary impact. The callus differentiation rate was pretty low (7.81%) without auxin. The differentiation rate was significantly increased with auxin supplementation. Therefore, we compared the combination of auxin supplementation with and without auxin. Consistent with the higher differentiation rate (70.83 vs. 42.86%) under 0.05 mg L^−1^ IAA treatment (Table 1), we analyzed the enriched metabolic pathways at 0.05 mg L^−1^ (C2) concentration treated by IAA. The results showed that when IAA was added, photosynthesis, brassinosteroid biosynthesis, and cell cycle pathways were enriched in C0-5D vs. IAA-C2-5D (Figure 4) at the embryogenic responsive stage (5 days). Pathways of phenylpropanoid biosynthesis and carbon metabolism were considerably enriched in the pre-embryonic initial period (20 days) (Figure 4). These results showed that photosynthesis, cell cycle, brassinosteroid biosynthesis, and phenylpropanoid biosynthesis pathways were essential to cotton SE under the condition of IAA treatment. The phenylpropanoid has extensive physiological activities related to plant growth regulation and disease-resistant ability.

##### Enriched Specific Pathway in IAA Dose Effect

**Figure 5:**
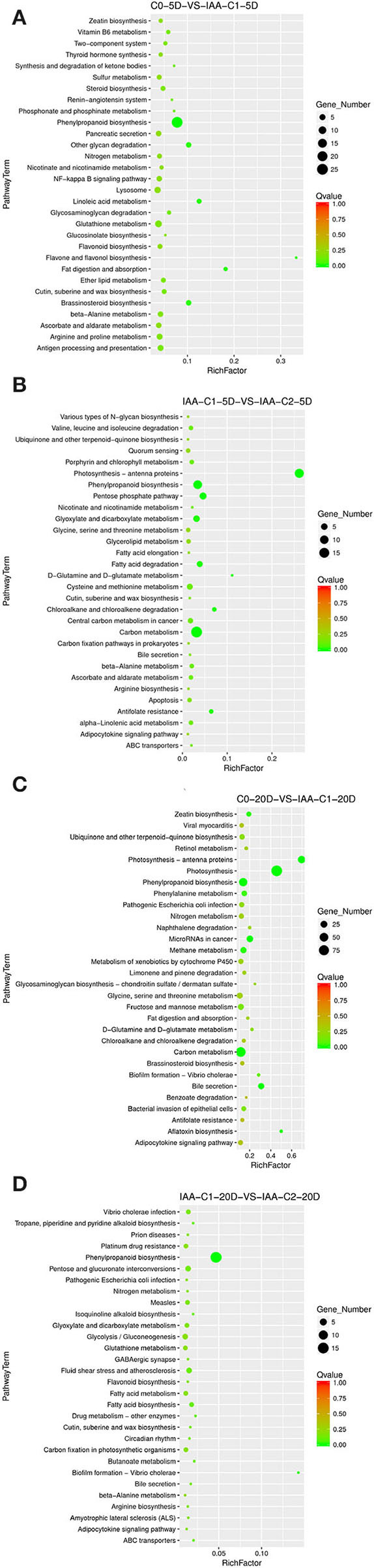
The KEGG enrichment of DEGs in comparison groups under the IAA dose effect. **(A)** Pathways enriched in C0-5D vs. IAA-C1-5D; **(B)** Pathways enriched in IAA-C1-5D vs. IAA-C2-5D; **(C)** Pathways enriched in C0-20D vs. IAA-C1-20D; and **(D)** Pathways enriched in IAA-C1-20D vs. IAA-C2-20D. IAA, indole acetic acid; 24D, 2,4-dichlorophenoxyacetic acid; C0, 0 mg L^−1^; C1, 0.025 mg L^−1^; C2, 0.05 mg L^−1^; 5D, 5 days' treatment; 20D, 20 days' treatment.

Different concentrations of IAA were added, and the differentiation rate of cotton callus was different. When the concentrations were 0.025 and 0.05 mg L^−1^, the high IAA concentration promoted differentiation. To analyze the IAA dose effect, we compared it with a combination of different concentrations of IAA. When IAA was added, the pathways significantly enriched were phenylpropanoid biosynthesis, photosynthetic-antennary proteins, pentose phosphate pathway, and the carbon metabolism pathway in the embryogenic responsive stage (5 days) (Figure 5). In the pre-embryonic initial period (20 days) (Figure 5), the biosynthetic pathways of phenylpropanoid were significantly enriched. It was speculated that the phenylpropanoid biosynthesis pathway affected the SE of cotton.

KEGG enrichment analysis indicated that photosynthesis, secondary metabolism, fatty acid metabolism, carbon metabolism, and phenylpropanoid biosynthesis pathways play a critical role in the cotton SE. We explored and analyzed DEGs involved in these KEGG pathways in cotton SE induced by IAA (Table 3).

**Table 3:** Representative DEGs induced by IAA.

**Gene ID**	**gene name**	**Gene Description**	**Pathway Annotation**	**Log_**2**_ (FC)**	**Comparison group**
Gh_A01G1294	*PSBY*	photosystem II core complex proteins psbY, chloroplastic	photosynthesis	−1.21	C0-5D-VS-IAA-C2-5D
Gh_A08G1679	*PSBW*	photosystem II reaction center W protein, chloroplastic-like	photosynthesis	−1.11	C0-5D-VS-IAA-C2-5D
Gh_D06G0548	*CYP734A1*	cytochrome P450 734A1-like	Brassinosteroid biosynthesis	−1.07	C0-5D-VS-IAA-C2-5D
Gh_A07G2172	*RBCS*	ribulose bisphosphate carboxylase small chain, chloroplastic isoform X1	Carbon metabolism	−3.64	C0-20D-VS-IAA-C2-20D
Gh_A05G1647	*DFR*	dihydroflavonol-4-reductase-like	Flavonoid biosynthesis	−1.01	C0-20D-VS-IAA-C2-20D
Gh_A01G1563	*CUT1*	3-ketoacyl-CoA synthase 6	Fatty acid elongation	2.43	C0-20D-VS-IAA-C2-20D
Gh_A09G1415	*PER21*	peroxidase 21	Phenylpropanoid biosynthesis	−2.02	C0-5D-VS-IAA-C1-5D
Gh_D08G0829	*PER73*	peroxidase 73	Phenylpropanoid biosynthesis	−1.93	C0-5D-VS-IAA-C1-5D
Gh_D12G1798	*CYP75B2*	Flavonoid 3′-monooxygenase	Flavonoid biosynthesis	−1.06	C0-5D-VS-IAA-C1-5D
Gh_Sca006141G01	*accD*	Acetyl-coenzyme A carboxylase carboxyl transferase subunit beta, chloroplastic	Pyruvate metabolism	−1.77	C0-5D-VS-IAA-C1-5D
Gh_A01G1681	*CYP98A2*	cytochrome P450 98A2-like	Flavonoid biosynthesis	−1.2	C0-20D-VS-IAA-C1-20D
Gh_D11G1768	*SEND33*	ferredoxin–NADP reductase, leaf isozyme, chloroplastic	photosynthesis	−2.75	C0-20D-VS-IAA-C1-20D
Gh_A01G1598	*PSBS*	photosystem II 22 kDa protein, chloroplastic-like	photosynthesis	−2.64	C0-20D-VS-IAA-C1-20D
Gh_D07G2129	*PSBW*	photosystem II reaction center W protein, chloroplastic-like	photosynthesis	−2.81	C0-20D-VS-IAA-C1-20D
Gh_A10G0948	*PPD*	pyruvate, phosphate dikinase, chloroplastic	Carbon metabolism	−1.26	IAA-C1-5D-VS-IAA-C2-5D
Gh_A10G2288	*PER27*	peroxidase 27-like	Phenylpropanoid biosynthesis	−2.2	IAA-C1-5D-VS-IAA-C2-5D
Gh_D08G0829	*PER59*	peroxidase 59	Phenylpropanoid biosynthesis	2.18	IAA-C1-20D-VS-IAA-C2-20D
Gh_A01G0158	*SERK1*	somatic embryogenesis receptor kinase 2-like isoform X1	–	1.34	24D-C3-0D-VS-C0-5D
Gh_A03G0840	*RAV1*	AP2/ERF and B3 domain-containing transcription factor RAV1-like	–	−1.19	24D-C3-0D-VS-IAA-C2-20D
Gh_D11G3261	*WOX8*	WUSCHEL-related homeobox 8-like	–	2.85	24D-C3-0D-VS-IAA-C1-5D
Gh_A11G0358	*ARF2*	auxin response factor 2 isoform X1	–	1.19	24D-C3-0D-VS-IAA-C1-5D
Gh_A01G0908	*ARF5*	auxin response factor 5 isoform X1	–	1.68	24D-C3-0D-VS-IAA-C1-5D
Gh_A12G0504	*LTP2*	non-specific lipid-transfer protein 2	–	1.34	24D-C3-0D-VS-IAA-C1-5D

*IAA, indole acetic acid; 24D, 2,4-dichlorophenoxyacetic acid; C0, 0 mg L^−1^; C1, 0.025 mg L^−1^; C2, 0.05 mg L^−1^; C3, 0.1 mg L^−1^; 5D, 5 days' treatment; 20D, 20 days' treatment*.

#### KEGG Enrichment Pathway Induced by 2,4-D

##### Enriched Specific Pathway Under the 2,4-D Induction Effect

**Figure 6:**
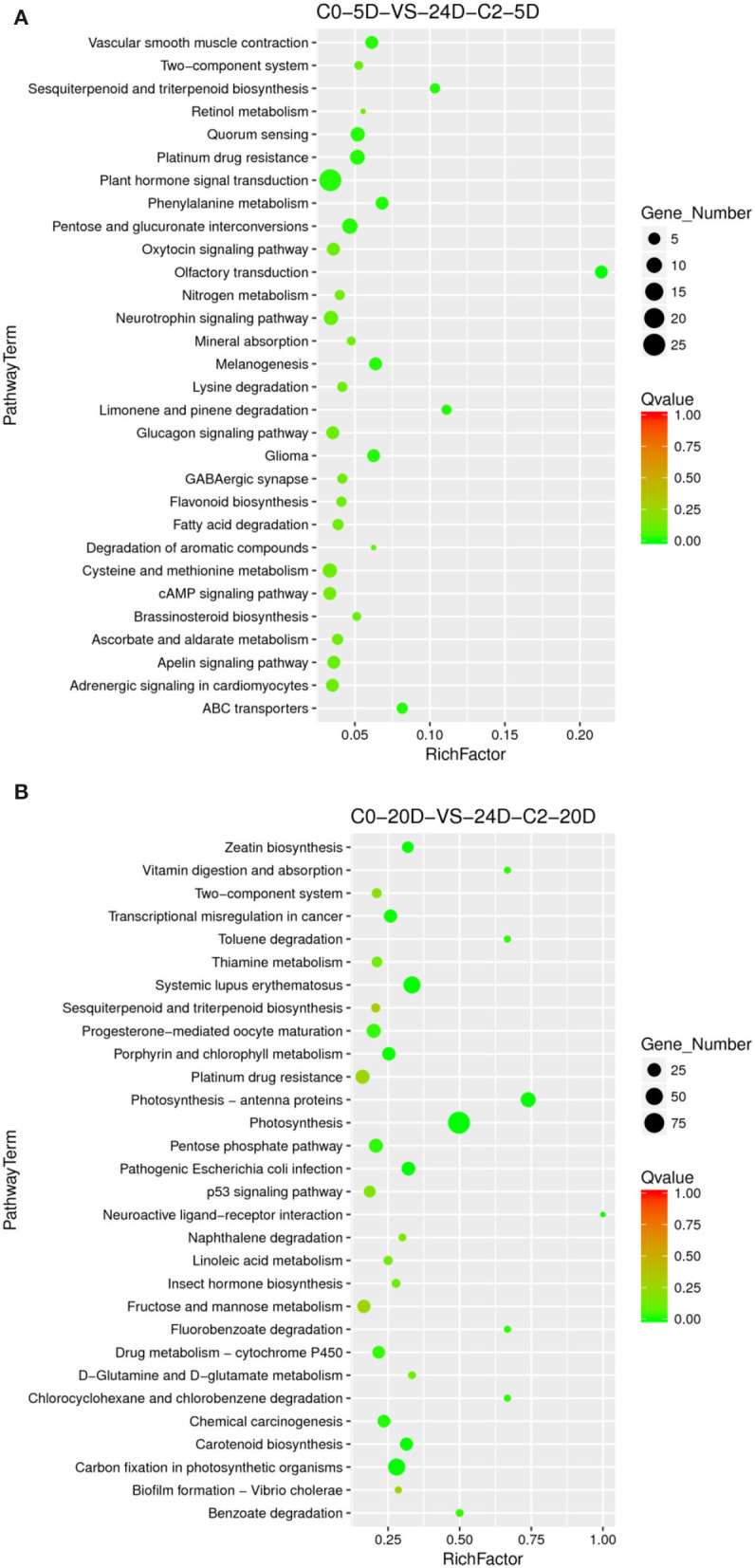
The KEGG enrichment of DEGs in comparison groups under the 2,4-D induction effect. **(A)** Pathways enriched in C0-5D vs. 24D-C2-5D; **(B)** Pathways enriched in C0-20D vs. 24D-C2-20D. 24D, 2,4-dichlorophenoxyacetic acid; C0, 0 mg L^−1^; C2, 0.05 mg L^−1^; 5D, 5 days' treatment; 20D, 20 days' treatment.

Compared to the phenotype, the cotton callus differentiation rate was significantly increased with 2,4-D addition. We compared the combination of supplementation with and without 2,4-D. Consistent with the higher differentiation rate (33.82 vs. 19.44%) under 0.05 mg L^−1^ 2,4-D treatment (Table 1), we analyzed the enriched metabolic pathways at 0.05 mg L^−1^ (C2) concentration treated by 2,4-D. The results demonstrated that the signal transduction pathway of plant hormone was highly enriched (Figure 6) in the embryogenic responsive stage (5 days) when 2,4-D was added. The photosynthesis-antennal proteins, photosynthesis, zeatin biosynthesis, and carbon fixation of photosynthetic organisms' pathways were enriched (Figure 6) in the pre-embryonic initial period (20 days).

##### Enriched Specific Pathway Under 2,4-D Dose Effect

**Figure 7:**
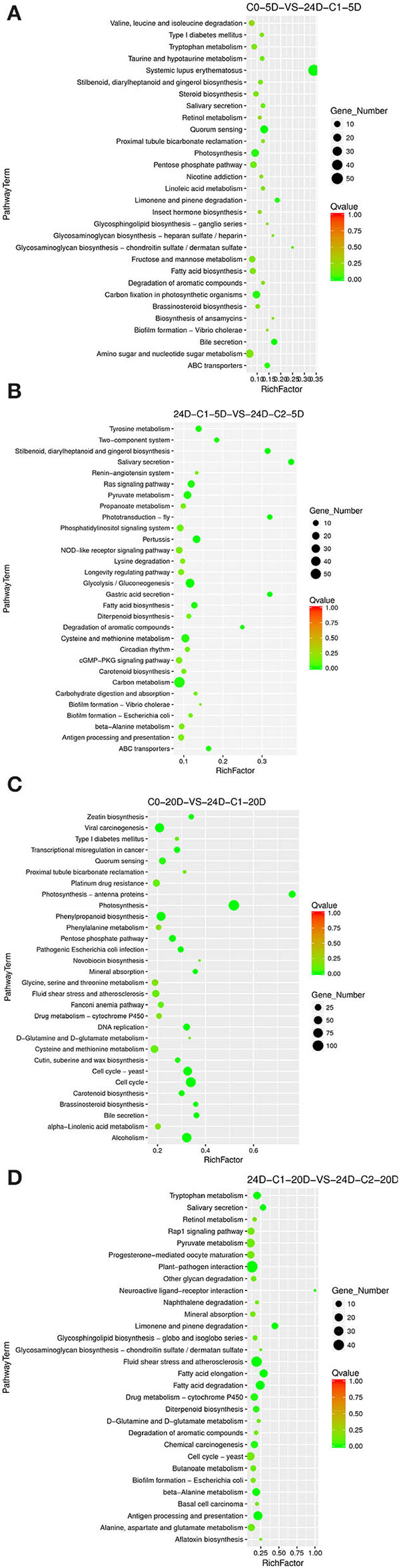
The KEGG enrichment of DEGs in comparison groups under 2,4-D dose effect. **(A)** Pathways enriched in C0-5D vs. 24D-C1-5D; **(B)** Pathways enriched in 24D-C1-5D vs. 24D-C2-5D; **(C)** Pathways enriched in C0-20D vs. 24D-C1-20D; **(D)** Pathways enriched in 24D-C1-20D vs. 24D-C2-20D. 24D, 2,4-dichlorophenoxyacetic acid; C0, 0 mg L^−1^; C1, 0.025 mg L^−1^; C2, 0.05 mg L^−1^; 5D, 5 days' treatment; 20D, 20 days' treatment.

Different 2,4-D concentrations were added, and the cotton callus differentiation rates differed. Compared to the concentrations between 0.025 and 0.05 mg L^−1^, the high concentration of 2,4-D promoted differentiation. To analyze 2,4-D dose effects, we compared various combinations of 2,4-D concentrations. When 2,4-D was added, photosynthesis, carbon metabolism, pyruvate metabolism, and ABC transporter pathways (Figure 7) were enriched in the embryogenic responsive stage (5 days). In the pre-embryonic initial period (20 days), photosynthesis, phenylpropanoid biosynthesis, cell cycle, alcohol degradation, fatty acid degradation, and fatty acid elongation pathways were enriched (Figure 7). It was speculated that primary metabolism and fatty acid metabolism affect the SE of cotton.

KEGG enrichment analysis indicated that the pathways of plant hormone signal transduction, carbon metabolism, secondary metabolism, photosynthesis, cell cycle, and fatty acid metabolism played a critical role in the cotton SE process. We explored and analyzed DEGs involved in these KEGG pathways in the cotton SE induced by 2,4-D (Table 4).

**Table 4:** Representative DEGs induced by 2,4-D.

**Gene ID**	**gene name**	**Gene Description**	**Pathway Annotation**	**Log_**2**_ (FC)**	**Comparison group**
Gh_D05G1217	*AUX22*	auxin-induced protein AUX22	plant hormone signal transduction	1.46	C0-5D-VS-24D-C2-5D
Gh_A01G1955	*LAX2*	auxin transporter-like protein 2	plant hormone signal transduction	1.08	C0-5D-VS-24D-C2-5D
Gh_D08G2503	*IAA1*	auxin-responsive protein IAA1-like	plant hormone signal transduction	1.43	C0-5D-VS-24D-C2-5D
Gh_D06G1764	*PYL4*	abscisic acid receptor PYL4-like	plant hormone signal transduction	−1.24	C0-5D-VS-24D-C2-5D
Gh_A11G0443	*GH3.1*	probable indole-3-acetic acid-amido synthetase GH3.1	plant hormone signal transduction	1.41	C0-5D-VS-24D-C2-5D
Gh_A12G2619	*SAUR32*	uncharacterized protein LOC105764246	plant hormone signal transduction	−1.47	C0-5D-VS-24D-C2-5D
Gh_D06G0548	*CYP734A1*	cytochrome P450 734A1-like	Brassinosteroid biosynthesis	−1.65	C0-5D-VS-24D-C2-5D
Gh_D09G0130	*CML45*	probable calcium-binding protein CML45	Calcium signaling pathway	1.66	C0-5D-VS-24D-C2-5D
Gh_A10G0948	*PPD*	pyruvate, phosphate dikinase, chloroplastic	Carbon metabolism	1.42	C0-20D-VS-24D-C2-20D
Gh_A01G1294	*PSBY*	photosystem II core complex proteins psbY, chloroplastic	photosynthesis	−2.51	C0-20D-VS-24D-C2-20D
Gh_A08G1679	*PSBW*	photosystem II reaction center W protein, chloroplastic-like	photosynthesis	−1.69	C0-20D-VS-24D-C2-20D
Gh_A01G1527	*LACS2*	long chain acyl-CoA synthetase 2 isoform X2	Fatty acid biosynthesis	−4.73	C0-5D-VS-24D-C1-5D
Gh_Sca006141G01	*accD*	Acetyl-coenzyme A carboxylase carboxyl transferase subunit beta, chloroplastic	Fatty acid biosynthesis	−2.01	C0-5D-VS-24D-C1-5D
Gh_A05G3293	*PSBP*	hypothetical protein B456_012G039400	Photosynthesis	1.01	C0-5D-VS-24D-C1-5D
Gh_D12G0245	*SEND33*	ferredoxin-2-like	Photosynthesis	1.61	C0-5D-VS-24D-C1-5D
Gh_D10G0167	*PSBQ2*	oxygen-evolving enhancer protein 3, chloroplastic	Photosynthesis	1.13	C0-5D-VS-24D-C1-5D
Gh_D12G0039	*psaD*	photosystem I reaction center subunit II, chloroplastic	Photosynthesis	1.82	C0-5D-VS-24D-C1-5D
Gh_A01G1527	*LACS2*	long chain acyl-CoA synthetase 2 isoform X2	Fatty acid biosynthesis	4.87	24D-C1-5D-VS-24D-C2-5D
Gh_Sca006141G01	*accD*	Acetyl-coenzyme A carboxylase carboxyl transferase subunit beta, chloroplastic	Fatty acid biosynthesis	1.2	24D-C1-5D-VS-24D-C2-5D
Gh_A09G0137	*CML45*	probable calcium-binding protein CML45	Calcium signaling pathway	2.06	24D-C1-5D-VS-24D-C2-5D
Gh_D07G1759	*RBCS*	ribulose bisphosphate carboxylase small chain, chloroplastic isoform X1	Carbon metabolism	−2.17	24D-C1-5D-VS-24D-C2-5D
Gh_A07G1821	*sna41*	cell division control protein 45 homolog	Cell cycle	2.14	C0-20D-VS-24D-C1-20D
Gh_D11G0102	*CDC20-1*	cell division cycle 20.2, cofactor of APC complex-like	Cell cycle	1.64	C0-20D-VS-24D-C1-20D
Gh_A01G1294	*PSBY*	photosystem II core complex proteins psbY, chloroplastic	photosynthesis	−2.8	C0-20D-VS-24D-C1-20D
Gh_A08G1679	*PSBW*	photosystem II reaction center W protein, chloroplastic-like	photosynthesis	−1.95	C0-20D-VS-24D-C1-20D
Gh_A06G1446	*CYP82A3*	cytochrome P450 82A3	Brassinosteroid biosynthesis	3.25	C0-20D-VS-24D-C1-20D
Gh_D11G0606	*ADH1*	alcohol dehydrogenase 1	Fatty acid degradation	2.49	24D-C1-20D-VS-24D-C2-20D
Gh_A01G1563	*CUT1*	3-ketoacyl-CoA synthase 6	Fatty acid elongation	4.07	24D-C1-20D-VS-24D-C2-20D
Gh_A05G0335	*CBL4*	calcineurin B-like protein 4 isoform X1	Calcium signaling pathway	1.48	24D-C1-5D-VS-24D-C2-5D
Gh_D05G0341	*ABP20*	auxin-binding protein ABP20-like	–	1.45	24D-C1-5D-VS-24D-C2-5D
Gh_A12G0175	*SERK2*	somatic embryogenesis receptor kinase 2-like	–	1.25	24D-C1-5D-VS-24D-C2-5D
Gh_A06G0222	*AP2/ERF*	AP2-like ethylene-responsive transcription factor ANT	–	1.65	C0-5D-VS-24D-C1-5D
Gh_A11G0886	*ARF18*	auxin response factor 18-like isoform X2	–	−1.94	C0-5D-VS-24D-C1-5D
Gh_A06G2038	*ARF3*	auxin response factor 3-like isoform X1	–	1.27	C0-5D-VS-24D-C1-5D
Gh_A05G1607	*ARF5*	auxin response factor 5 isoform X2	–	1.14	C0-5D-VS-24D-C1-5D
Gh_A08G2008	*BBM*	AP2-like ethylene-responsive transcription factor BBM	–	1.61	24D-C3-0D-VS-24D-C1-20D
Gh_A01G0393	*RAV1*	AP2/ERF and B3 domain-containing transcription factor RAV1-like	–	2.12	24D-C3-0D-VS-24D-C2-20D
Gh_D05G1962	*WOX4*	WUSCHEL-related homeobox 4-like	–	2.31	24D-C3-0D-VS-24D-C1-5D
Gh_A07G1254	*ARF18*	auxin response factor 18 isoform X1	–	−1.67	24D-C3-0D-VS-24D-C1-5D
Gh_D05G0755	*ARF4*	auxin response factor 4 isoform X1	–	4.31	24D-C3-0D-VS-24D-C1-5D
Gh_D12G2535	*LTP2*	non-specific lipid-transfer protein 2-like	–	3.44	24D-C3-0D-VS-24D-C1-5D

*24D, 2,4-dichlorophenoxyacetic acid; C0, 0 mg L^−1^; C1, 0.025 mg L^−1^; C2, 0.05 mg L^−1^; C3, 0.1 mg L^−1^; 5D, 5 days' treatment; 20D, 20 days' treatment*.

#### Induction Effect and Dose Effect of Different Auxin Treatments by IAA and 2,4-D

The cotton callus differentiation rate showed that auxin affects the differentiation rate highly. Without auxin, the callus differentiation rate was pretty low (7.81%), and the differentiation rate was significantly increased with auxin supplementation. Therefore, we compared the combination of auxin supplementation with and without auxin.

In the embryogenic response stage (5 days), signal transduction of the plant hormone pathway was significantly enriched under 2,4-D induction. The auxin signal genes, including *AUX, IAA, LAX, ARF, PYL*, and *GH3*, were differentially expressed in the signal transduction pathway (Fan et al., 2020). The results indicate that the genes related to auxin signal transduction are necessary for the initial response of embryogenic differentiation. In the pre-embryonic initial period (20 days), a photosynthetic pathway was significantly enriched under the induction of 2,4-D. The DEGs (*PSBS, PSBQ2, SEND33*, and *PSBW*) were significantly enriched in this pathway, and most of the genes were downregulated (Tables 3, 4). These results suggest that the photosynthesis pathway may play a negative role in the early stage of somatic embryogenesis. The photosynthesis and cell cycle pathways were strikingly enriched in the embryogenic response stage (5 days) on the condition of IAA induction. The carbon metabolism pathways were considerably enriched in the pre-embryonic initial period (20 days). Photosynthesis and cell cycle pathways were enriched during the response stage of embryogenic induction under IAA treatment. Therefore, the IAA may have different somatic embryo induction mechanisms to 2,4-D.

Different auxin concentrations were added to induce cotton callus differentiation. When the concentrations were 0, 0.025, and 0.05 mg L^−1^, a high concentration of 2,4-D and IAA promoted differentiation. To analyze the dose effect of various auxins, we compared KEGG-enriched pathways under different auxin concentrations.

In the embryogenic response stage (5 days), compared with two concentrations of IAA and 2,4-D, results showed that the DEGs were strikingly enriched in the carbon metabolism pathway. Compared with the two concentrations of IAA, the phenylpropanoid biosynthesis pathway was dose-dependent. The results showed that the secondary metabolism and phenylpropanoid biosynthesis pathway affected somatic embryogenesis. The phenylpropanoid biosynthesis pathway was considerably enriched in different stages under the IAA dose effect, and it was significantly enriched in the pre-embryonic initial period (20 days) under the 2,4-D dose effect. The *PER* gene related to the phenylpropanoid synthesis pathway was differentially expressed. The peroxidase was encoded by *PER* gene. The peroxidase regulated the peroxidase protein activity during somatic embryogenesis by controlling the callus browning and then affected somatic embryogenic differentiation. Upon auxin dose effect, the fatty acid metabolism pathway (including fatty acid elongation, fatty acid biosynthesis, and fatty acid degradation) was enriched significantly under 2,4-D and IAA treatments. The fatty acid is speculated to provide energy for somatic embryo development and affect cell growth and differentiation.

#### Confirmation of Candidate Gene Expression Patterns

**Figure 8:**
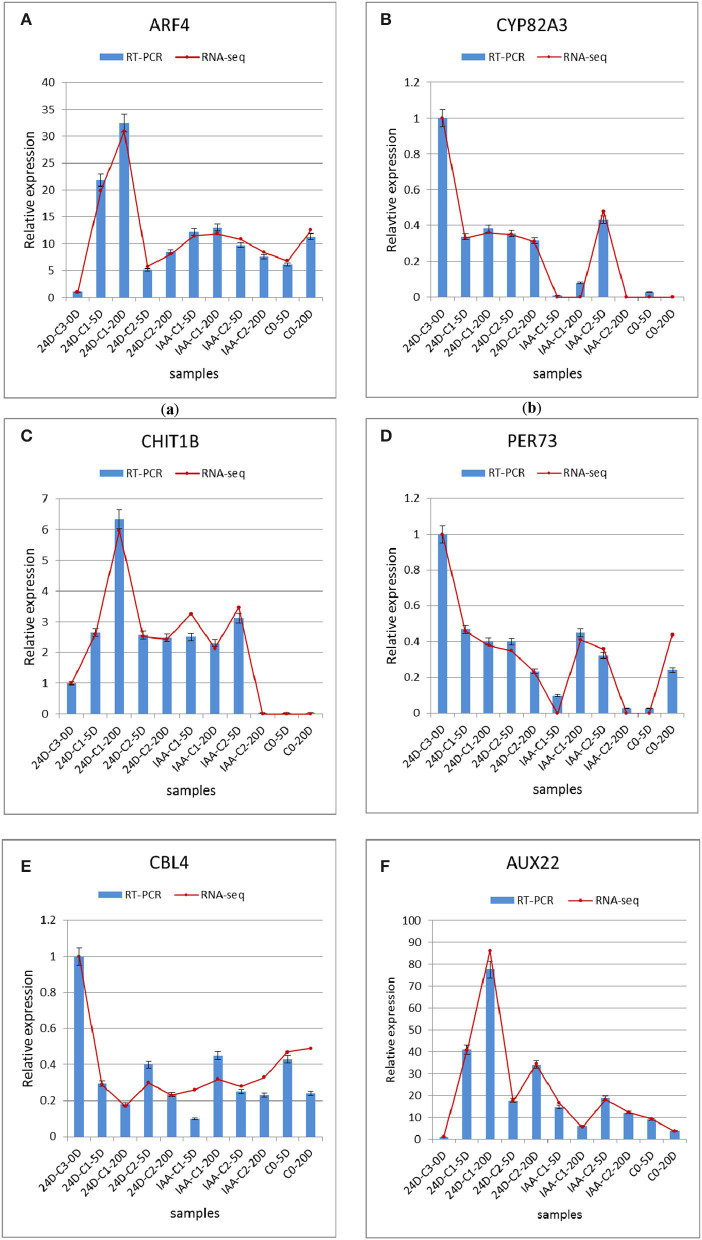
Comparison and confirmation of the RNA-seq data with qRT-PCR. **(A–F)** Relative expression of DEGs selected by comparative transcriptome induced by IAA and 2,4-D. Bars represent SD, and gene abundance was calculated relative to the *GhUB7* expression level. IAA, indole acetic acid; 24D, 2,4-dichlorophenoxyacetic acid; C0, 0 mg L^−1^; C1, 0.025 mg L^−1^; C2, 0.05 mg L^−1^; C3, 0.1 mg L^−1^; 5D, 5 days' treatment; 20D, 20 days' treatment.

We used the qRT-PCR method to verify the DEGs analyzed in the expression profile. Six genes were selected to verify the results. The six genes were cytochrome P450 (*CYP82A3*), endochitinases (*CHIT1B*), peroxidase (*PER73*), calcineurin B-like protein (*CBL4*), auxin-induced protein (*AUX22*), and auxin response factor (*ARF4*) (Fan et al., 2020). These genes' functions related to SE are detailed in the previous article (Fan et al., 2020). Auxin response factors (*ARFs*) are classic genes related to SE that are activated explicitly in the embryogenic differentiation courses (Passardi et al., 2007; Zhai et al., 2016; Guo et al., 2019a,c). *ARFs* and *Aux/IAAs* are the critical transcription factors regulating auxin-responsive gene expression. The auxin response factors (*ARFs*) known as gene families were studied by predecessors (Ulmasov et al., 1997; Okushima et al., 2005). *ARFs* mediate transcription responses to auxin by binding to auxin response promoter elements, then *Aux/IAA* regulates auxin-mediated transcription activation/inhibition (Lin et al., 2015; Su et al., 2016). Peroxidase proteins affect cotton SE by involving the process of phenylpropanoid biosynthesis (Cao et al., 2017). Peroxidase's function is related to many plant processes, including cell wall elongation, auxin metabolism, and hardening (Passardi et al., 2007). In our study, peroxidase was involved in the phenylpropanoid biosynthesis pathway. Peroxidase was interested in various metabolic pathways to regulate SE. Three repeated biological experiments have been carried out. The results are presented in Figure 8. The qRT-PCR results were in harmony with RNA-seq gene expression patterns, which indicated that transcriptome data were credible, and these genes were modulated in cotton auxin-induced SE. Their functions would be mined in further research.

## Discussion

Somatic embryogenesis (SE) is a seriously complicated biological process influenced by genotype, hormones, mineral elements, stress, light, nitrogen source, carbon source, and other factors. On internal and external factors, somatic cells transdifferentiate into embryogenic cells with the expression of some specific genes. Comparative transcriptome analysis helps reveal the SE underlying molecular mechanism. In this study, the changes in endogenous gene expression were studied by exogenous hormone treatment, trying to find the endogenous factors that responded to the changes in the exogenous hormone, which provided a reliable theoretical and experimental basis for the study of cotton SE.

### Auxin Types and Concentrations Affected Cotton Callus Embryogenic Differentiation

The experiment studied the differentiation effect of medium supplemented with different hormone combinations on cotton SE. IBA-induced transcriptome profiling has been reported in our previous study (Fan et al., 2020). In different hormone types (IAA and 2,4-D), different doses (0, 0.025, and 0.05 mg L^−1^), and different culture phases (0, 5, and 20 days), the induced gene transcription and expression profiles were compared and analyzed by high-throughput sequencing technology in cotton SE. We found that auxin types and concentrations had a further impact on embryogenic differentiation. In this experiment, the higher embryogenic differentiation rate was induced in the medium with the addition of 0.05 mg L^−1^ IAA + 0.10 mg L^−1^ KT. The lowest one was in the medium without auxin (0 mg L^−1^). The changes in callus embryogenic differentiation rate induced by exogenous auxin and cytokinin were in accordance with previous research. The embryogenic callus (EC) and somatic embryo (SE) induction were related to the 2,4-D concentration, which decreased to a certain level or was substituted by the combination of IBA and KT in the medium (Sun et al., 2006; Kumar et al., 2015). Zhu and Sun (2000) found that callus cultured with IAA, 2,4-D, and KT simultaneously had a better effect. At the same time, callus induced by 2,4-D had to be transferred to the medium with 2,4-D replaced or removed to yield SE. In this experiment, the concentration of 2,4-D decreased from 0.1 to 0.05 mg L^−1^, which was beneficial to the callus differentiation. The auxin and cytokinin's SE induction function has been found to interact with different pathways or components related to somatic embryo (SE) differentiation, including their upstream transcription regulatory factors (TFs) and hormonal signaling pathways (Cheng et al., 2016). Wang et al. (2004) found that the IBA effect was more practical than another auxin in cotton callus culture, consistent with our research results. Compared with 0.025 and 0.05 mg L^−1^, the higher IAA and 2,4-D concentrations promoted callus differentiation. These results indicated that proper auxin concentration was beneficial to callus differentiation. The embryogenic differentiation rate was the lowest in the medium with 0.1 mg L^−1^ KT, which indicated that the cytokinin alone inhibited cell differentiation. It is essential to add auxin and cytokinin appropriately during callus culture.

### Plant Hormone Signal Transduction Pathway Was Related to Cotton SE Under Auxin Induction Effect on Cotton Somatic Embryogenic Differentiation

Somatic embryo formation is a complex interaction process among medium, explant, culture environment, and genotype (Cui and Dai, 2000). The type, concentration, and proportion of plant growth regulators (PGRs) are dominant factors in modulating the callus embryogenic differentiation, which plays a crucial role in somatic embryo formation. SE induction is a complicated multifactor system relating endogenous and exogenous hormones, mainly stimulated by exogenous plant regulators (Jimenez, 2005).

Comparative transcriptome analysis helped to reveal the molecular mechanisms of SE. The transcriptome sequence analysis was implemented using high-throughput sequencing technology. The differentiation ability of cotton callus was influenced in two aspects, including *in vitro* and *in vivo*. The changes in endogenous genes in cotton callus were detected by exogenous hormone treatment for our study. The mechanism of endogenous genes responding to the changes in exogenous auxin was dug to provide an authentic theoretical and experimental foundation for cotton SE. The results showed that different genes were upregulated or downregulated under different auxin combinations. In the embryogenic responsive stage (5 days), more responsive genes participating in the SE progress were conducive to differentiation. In the pre-embryonic initial phase (20 days), a less number of corresponding differential genes was beneficial to the differentiation. The different concentrations of the same hormone had other differentiation effects. Higher 2,4-D and IAA concentrations have an advantage in callus differentiation.

The cotton callus embryogenic differentiation rates showed that the differentiation rate was the lowest without auxin. Therefore, the addition of auxin was essential for the embryogenic differentiation of cotton callus. Auxin had an induction effect on the embryogenic differentiation of cotton callus. To identify the induction effect of different auxins, we analyzed the KEGG enrichment pathways with or without auxin combination when the induction effect was better. The results showed that a pathway to plant hormone signal transduction was considerably enriched under 0.05 mg L^−1^ 2,4-D in the embryogenic responsive stage (5 days). Photosynthesis and cell cycle pathways were significantly enriched under 0.05 mg L^−1^ IAA. In the pre-embryonic initial stage (20 days), a photosynthetic pathway was significantly enriched under 0.05 mg L^−1^ 2,4-D. In contrast, a carbon metabolism pathway was enriched considerably under the 0.05 mg L^−1^ IAA.

Plant hormone signal transduction, photosynthesis, cell cycle, carbon metabolism, and other pathways are crucial in somatic embryo induction in cotton. The DEGs of the cell cycle pathway are enriched and upregulated, which indicates that the cell cycle may play a positive role in SE regulation. In the initial stage of proembryo formation, SE depends on a series of complex interactions between different PGRs, including auxin alone or combined with different cytokinins. In most of the species studied, PGR supplementation is essential to induce SE. Auxin and cytokinin are critical factors of SE, which may be due to their participation in cell division and cell cycle regulation (Kumar and Van Staden, 2017). Auxin can induce SE of cotton, and different auxins may have other inductive mechanisms.

Compared to the C0 combination (0 mg L^−1^), with the corresponding high differentiation rate of 2,4-D supplementation, the DEGs (*PSBS, PSBQ2, SEND33*, and *PSBW*) were significantly enriched in the photosynthetic pathway during the pre-embryonic initial stage (20 days) (Tables 3, 4) (Fan et al., 2020). Most of the genes in this pathway were downregulated. It suggested that photosynthesis might play a negative role in SE regulation. However, pathways to photosynthesis and the cell cycle were strikingly enriched during the embryogenic responsive period (5 days) at a higher differentiation rate concentration (0.05 mg L^−1^ IAA). The photosynthesis and cell cycle pathways affect cotton callus differentiation, especially in the embryogenic responsive stage. These results indicated that the induction mechanism of IAA was different from that of 2,4-D.

Exogenous hormones are necessary for the SE of cotton (Wolters and Jurgens, 2009). Among them, auxin was the chief plant growth regulator during the development of SE, which participated in cell division and differentiation regulation. It suggested that adding plant hormones to the medium had an extreme effect on the embryogenic differentiation rate. During the embryogenic responsive stage (5 days), the signal transduction of the plant hormone pathway was significantly enriched under 0.05 mg L^−1^ 2,4-D. *Aux, ARF, PYL, GH3*, and other auxin-associated genes were expressed differentially in this signal transduction pathway. *GH3.1* (indole-3-amido synthetase *GH3.1*) was upregulated in C0-5D vs. 24D-C2-5D. *ARF5* was upregulated, and *PYL4* was downregulated. Auxin-associated genes are necessary for the initiation of embryonic differentiation. The *GH* gene was expressed rapidly and specifically during auxin induction (Hagen and Guilfoyle, 1985; Yang and Zhang, 2010), which suggested that 2,4-D initiated signals by receptor binding and transduced signals by complicated downstream molecular events and realized signal output by inducing a response in cell morphology and metabolism. Exogenous 2,4-D signals were recognized by receptors, which trigger intracellular signal transduction and amplification, ultimately leading to cell and organ changes. Hormone tiny molecules, signal transduction, and response affect plant growth and development. These factors are necessary to cotton SE.

### Phenylpropanoid Biosynthesis and Fatty Acid Metabolism Pathway Affected SE Under Auxin Dose Effects on Cotton Somatic Embryogenic Differentiation

We summarized that the different auxin concentration combinations had various embryogenic differentiation effects according to the cotton callus embryogenic differentiation rate. The results demonstrated that the embryogenic differentiation rate was higher in the medium with the addition of 0.05 mg L^−1^ IAA + 0.10 mg L^−1^ KT than that treated with 0.025 mg L^−1^ 2,4-D + 0.10 mg L^−1^ KT (Table 1). Under the same auxin concentration, high 2,4-D and IAA concentrations promoted callus differentiation. Transcriptome sequencing data showed that DEGs were significantly enriched in the carbon metabolism pathway compared between two concentrations of IAA and 2,4-D during the embryogenic responsive stage (5 days) (Figures 5B, 7B). Analyzing different IAA and 2,4-D combinations of 0, 0.025, and 0.05 mg L^−1^, we found that the phenylpropanoid biosynthesis pathway had a dose-dependent effect under IAA treatment. These results suggest that phenylpropanoid biosynthesis and secondary metabolism pathways affect SE in cotton.

Zhang et al. (1992) found that the secondary metabolites' content, including anthocyanin, chlorophyll, total phenols, and flavonoids, induced in embryogenic callus was lower than those in non-embryogenic callus in cotton embryogenesis. Sun et al. (2018) also found that in the comparative transcriptome study of cotton SE, the active secondary metabolites blocked the primary metabolism of recalcitrant CCRI12 materials, resulting in delayed cell differentiation. These studies showed that the secondary metabolites in embryonic callus were lower than those in non-embryonic callus. The results indicated that the secondary metabolism levels in the non-embryonic callus were higher, and the secondary metabolism affected cell differentiation by affecting the rate and intensity of the primary metabolism. Meanwhile, the biosynthesis of phenylpropanoid may play a critical role in SE in our research.

The phenylpropanoid biosynthesis pathway was strikingly enriched at different stages (5 and 20 days) under the IAA dose effect. This pathway was remarkably enriched in the pre-embryonic initial phase (20 days) of the 2,4-D dose effect. Phenylpropanoid had a wide scale of physiological activities and was related to plant growth regulation. The *PER* gene involved in phenylpropanoid synthesis was differentially expressed. The peroxidase encoded by *PER* was a highly active enzyme that existed in most plants. It was related to photosynthesis, respiration, and the oxidation of auxin. Its activity was constantly changing during plant growth and development. Generally, the activity of aging tissues was higher; however, the activity of young tissues was weaker. Peroxidase participated in many plants' growth and development, such as cell wall elongation, auxin metabolism, and hardening (Passardi et al., 2007; Guo et al., 2019b). Our results suggested that peroxidase was mainly related to the phenylpropanoid biosynthesis pathway (Figures 5, 7C, Table 3) (Fan et al., 2020). Peroxidase was a phenolic oxidase, which was significantly represented in date palms, and polyphenol oxidase was essential in the oxidative browning of date palms (Baaziz and Saaidi, 1988; Baaziz, 1989). Abohatem et al. (2011) showed that the low-rate continuous transfer cultures decreased the phenolic content and peroxidase activities in plant tissues, leading to increased tissue/cell browning, and reducing embryonic cell proliferation. According to the transcriptional data, the peroxidase gene regulates the activity of peroxidase protein, which controls the browning process of callus during cotton SE, and then affects the embryogenic differentiation of somatic cells. We speculated that the peroxidase regulates SE in cotton callus through participating in the phenylpropanoid biosynthesis.

The pathways between the two concentrations showed that the fatty acid metabolism (including fatty acid synthesis, fatty acid elongation, and fatty acid degradation) pathway was significantly enriched under IAA and 2,4-D treatment, respectively. As a protective chemical substance, fatty acids played a crucial role in cotton's development and evolution. Fatty acids were the main structural components for triacylglycerol storage oils and membrane phospholipids in plants. In cotton SE, genes related to fatty acid syntheses such as *LACS2, ADH1, CUT1*, and *accD* were differently expressed. It indicated that genes related to fatty acid metabolism were differentially expressed during somatic embryo development, which might provide energy for somatic embryo development. Fatty acids played a crucial role in regeneration induction by affecting cell function and development (Guo et al., 2020).

### Screening Analysis of Classic Genes During Cotton SE

Several classic genes have been proven in experiments to play crucial roles in SE (Santos et al., 2005; Passardi et al., 2007; Zeng et al., 2007a; Cao et al., 2017). In the analysis of transcriptome data, we also found that *SERK, ARF, ABP, BBM, AP2/ERF, WOX, IAA, AUX, LTP*, and other critical genes related to SE (Tables 3, 4). Schmidt et al. (1997) discovered that *SERK1* was identified during hypocotyl regeneration in carrots, which regulated somatic cell transition to embryonic cells. In our previous research (Fan et al., 2020), *GhSERK1* (GH_A01G0158) had been discovered, and it was upregulated. The results suggested that *SERK* was essential in cotton SE induction.

The experiments showed that *BABY BOOM* (*BBM*) gene encoding the *AP2/ERF* family was related to the molecular regulation of SE, microspore embryogenesis, and zygotic embryogenesis. Setting the 24D-C3-0D sample as control, *AP2/ERF* and B3 domain transcription factors were downregulated in IAA-C2-20D and 24D-C1-20D, while they were upregulated in 24D-C2-20D. There was no significant differential expression in IAA-C1-20D. In the pre-embryonic initial stage (20 days), the upregulated expression of *AP2/ERF* and B3 domains was conducive to embryogenesis, suggesting that these transcription factors played a positive regulatory part in cotton SE.

We found that the WUSCHEL-related homeobox *WOX* gene was induced. *GhWOX4* and *GhWOX8* were significantly differentially expressed in all comparison groups at 5 and 20 days. These results indicate that *GhWOX* plays an essential role in auxin-induced cotton SE. Our research found that the auxin response factor (*ARF*) was expressed in all samples. In all comparison groups, the expressions of *ARF8* and *ARF18* were downregulated. Otherwise, the expression of *ARF2, ARF3, ARF4*, and *ARF5* was upregulated. The results showed that *ARFs* were involved in auxin-induced cotton SE regulation. So far, 23 *ARFs* genes have been isolated. Most *ARF* genes act by activating gene transcription, except *ARF3, ARF5, ARF7*, and *ARF8, which* act as transcriptional suppressors (Ren et al., 2012). The lipid transfer protein *LTP2* gene was differentially expressed in all the comparison combinations, and most of the expressions were upregulated. Guo et al. (2019d) found that SELTP drove cotton somatic totipotency and was a critical gene in cotton SE.

## Conclusion

Transcriptome sequencing provides a powerful tool to identify gene expression patterns during SE development. We used RNA-Seq to investigate gene expression patterns to find critical genes that regulate the switch gene response to the different auxin concentrations and hormone combinations during the SE. By analyzing large-scale transcriptome data, we have identified some genes related to embryogenesis and clarified the crucial regulatory role of some transcription factors in SE. Further research is required to expend on the consequences obtained to determine the molecular mechanisms supporting the complex gene expression patterns of the redifferentiation embryogenic initiation.

## Data Availability Statement

The data presented in the study are deposited in the NCBI repository, accession number is SRP379841.

## Author Contributions

FZ, YuF, and HuG conceived and designed the research project. YuF, JW, XY, HuG, YiF, and CZ performed tissue and cell sampling. YuF, ZT, XY, JW, HuG, TL, HaG, and LZ performed all morphological, molecular, and expression experiments. YF, ZT, HuG, and FZ analyzed the data and wrote the article. All authors contributed to the article and approved the submitted version.

## Funding

This study was supported by the Taishan Scholar Talent Project (TSQN20161018), Anhui Natural Science Research Project (KJ2020B26) equally, and by the National Natural Science Foundation of China (31401428).

## Conflict of Interest

The authors declare that the research was conducted in the absence of any commercial or financial relationships that could be construed as a potential conflict of interest.

## Publisher's Note

All claims expressed in this article are solely those of the authors and do not necessarily represent those of their affiliated organizations, or those of the publisher, the editors and the reviewers. Any product that may be evaluated in this article, or claim that may be made by its manufacturer, is not guaranteed or endorsed by the publisher.

## Abbreviations

2,4-D: 2,4-Dichlorophenoxyacetic acid
ARF: Auxin response factor
DEG: Differentially expressed gene
DGE: Digital gene expression
EC: Embryogenic callus
ERF: Ethylene response factor
GO: Gene ontology
IAA: Indole acetic acid
KEGG: Kyoto encyclopedia of genes and genomes
KT: Kinetin
MS: Murashige and Skoog
NAA: Naphthalene acetic acid
NR: Non-redundant Protein Sequence Database in GenBank
PGR: Plant growth regulator
RPKM: Reads Per Kilo bases per Million reads
RT-PCR: Reverse-transcription-polymerase chain reaction
SE: Somatic embryogenesis
*SERK*: Somatic embryogenesis receptor kinase.

## Appendix A

**Table A1:** Primer sequence for qRT-PCR.

**Gene**	**Full name**	**Primer sequence (Forward/Reverse)**
*CHIT1B*	Basic endochitinase	AGCAGGAGAAGCAATAAAGG/ATGACACCGTATCCAGGGAC
*CBL4*	Calcineurin B-like protein 4	TCTTGCTGCTGAAACACCT/TCTAACGAACTCACCGAAC
*PER73*	Peroxidase 73	GCAACTATCCGCCTCTTCT/GCTTAACCCATCCAACCTC
*CYP82A3*	Cytochrome P450 82A3	CTTTGGTAGTGGTAGGAGGAG/GAGCAAGACGAGGTGAGAC
*ARF4*	Auxin response factor 4	CAACATGAATCAAGCACCCAA/GTTTCCTGATGTCGTCCAC
*AUX22*	Auxin-induced protein 22	GGCAACTCTTCGGAGCAAC/TATCCCACTAATTTCACCC
*GhUB7*	Ubiquitin 7	GAAGGCATTCCACCTGACCAAC/CTTGACCTTCTTCTTCTTGTGCTT
